# Wnt Binding Affinity Prediction for Putative Frizzled-Type Cysteine-Rich Domains

**DOI:** 10.3390/ijms20174168

**Published:** 2019-08-26

**Authors:** Mark Agostino, Sebastian Öther-Gee Pohl

**Affiliations:** 1 School of Pharmacy and Biomedical Sciences, Curtin Health and Innovation Research Institute, Curtin University, Perth WA 6845, Australia; 2 Curtin Institute for Computation, Curtin University, Perth WA 6845, Australia; 3 The Institute of Genetics and Molecular Medicine, Edinburgh Cancer Research Centre, University of Edinburgh, Edinburgh, EH4 2XU, UK

**Keywords:** Wnt signalling, Frizzled, smoothened, cysteine-rich domain, protein‒protein interactions

## Abstract

Several proteins other than the frizzled receptors (Fzd) and the secreted Frizzled-related proteins (sFRP) contain Fzd-type cysteine-rich domains (CRD). We have termed these domains “putative Fzd-type CRDs”, as the relevance of Wnt signalling in the majority of these is unknown; the RORs, an exception to this, are well known for mediating non-canonical Wnt signalling. In this study, we have predicted the likely binding affinity of all Wnts for all putative Fzd-type CRDs. We applied both our previously determined Wnt‒Fzd CRD binding affinity prediction model, as well as a newly devised model wherein the lipid term was forced to contribute favourably to the predicted binding energy. The results obtained from our new model indicate that certain putative Fzd CRDs are much more likely to bind Wnts, in some cases exhibiting selectivity for specific Wnts. The results of this study inform the investigation of Wnt signalling modulation beyond Fzds and sFRPs.

## 1. Introduction

The Wnt signalling pathway is an evolutionarily conserved signalling cascade that dictates many aspects of development and cell fate. It is initiated through the extracellular binding of Wnt ligands to membrane receptor complexes [1]. These signalling cascades are classified as canonical (β-catenin dependent) or non-canonical (β-catenin independent). β-catenin-dependent signalling is initiated following Wnt ligand binding to the cysteine-rich domain (CRD) of Frizzled (Fzd) receptors to recruit the low-density lipoprotein receptor 5/6 (LRP5/6) [2]. The LRP/Fzd receptor complex transduces signals to a cytoplasmic protein complex composed of glycogen synthase kinase 3β (GSK3β), casein kinase 1α (CK1α), Axin and adenomatous polyposis coli (APC). The stability of this destruction complex regulates the degradation and subcellular localisation of β-catenin, controlling Wnt-mediated transcriptional activity. Non-canonical pathways include the Wnt/Ca2+-mediated signalling pathway as well as the planar cell polarity (PCP) pathway, which influences cytoskeletal rearrangements and actin polymerisation [3]. Wnt ligands are lipid-modified with palmitoleic acid by the *O*-acyltransferase Porcupine [4]. This lipidation has been demonstrated to be essential for secretion and binding to Fzd receptors [5]. Wnt signalling is known to be negatively regulated by sequestration of Wnt ligands by endogenous secreted Frizzled-related proteins (sFRPs) [6,7], which carry a Fzd-type CRD thought to mediate this process.

A range of proteins other than the Fzds and sFRPs feature Fzd-type CRDs [8]. These include the membrane Frizzled-related protein (MFRP), carboxypeptidase Z (CPZ), atrial natriuretic peptide-converting enzyme (CORIN), collagen XVIII α-chain (COL18A1), smoothened (SMO), the muscle skeletal receptor tyrosine-protein kinase (MuSK), and the receptor tyrosine kinase-like orphan receptors (ROR1 and ROR2). With the exception of ROR1 and ROR2, which are known to be key receptors in the Wnt PCP pathway [9,10,11,12], the relevance of these proteins to Wnt signalling is largely unknown or incompletely proven; hence, we refer to the CRDs of these proteins as “putative Fzd-type CRDs.”

MFRP is a CRD-containing protein with a role in ophthalmological development and various pathologies [13,14,15]. There has yet to be any conclusive evidence of its role in Wnt signalling or carcinogenesis. CPZ is an enzyme that removes C-terminal amino acids, in particular arginine residues. It differs from other members of the carboxypeptidase family due to its N-terminal Fzd-like CRD [16]. CPZ has been demonstrated to act as a Wnt pathway agonist through its CRD-mediated interaction with Wnt4. Furthermore, CPZ has been shown to remove the C-terminal arginine of Wnt4a to promote canonical Wnt signalling [17]. A definitive role in cancer has yet to be elucidated, although genome-wide association studies (GWAS) studies have indicated a variant of the CPZ gene is associated with neuroblastoma tumorigenesis [18]. CORIN, is a serine protease that cleaves the pro-atrial natriuretic peptide (pro-ANP) to ANP. It contains two Fzd-type CRDs, the specific functions of which are currently unknown. CORIN can indirectly negatively regulate Wnt/β-catenin signalling by increasing levels of ANP, which reduces Wnt1-mediated β-catenin nuclear localisation in colon adenocarcinoma cells [19]. COL18A1, or endostatin, is a basement membrane protein, which has been implicated in tumour growth and angiogenesis [20]. It contains an N-terminal domain that shares homology with the Fzd CRD [21]. Proteolytic cleavage of the COL18A1 CRD results in the antagonism of Wnt3a-induced canonical Wnt signalling [22], indicating a clear role for COL18A1 in regulating Wnt signalling.

SMO is a key regulator of Hedgehog signalling, and free lipids, such as cholesterol, have been shown to trigger signalling through SMO [23,24]. However, protein agonists or antagonists for SMO have not been identified. Considerable crosstalk between Hedgehog and Wnt/β-catenin signalling has been demonstrated, with key regulatory enzymes of the canonical Wnt pathway, GSK3β and CK1α, both negatively regulating Hedgehog/Gli signalling [25]. MuSK is a well-characterised receptor expressed in the neuromuscular synapses that regulates acetylcholine receptor (AChR) pre-patterning. Its association with Wnt signalling has been well studied in this context, with direct interactions occurring between Dvl1 and LRP4, which directly regulate the clustering of neurotransmitter receptors [26,27]. MuSK has also been shown to interact with wnt11r and wnt4a in the zebrafish through its CRD to regulate AChR signalling and MuSK endocytosis [28,29].

We have recently developed a computational model for predicting the binding energy (and hence, binding affinity) for Wnt interactions with Fzd-type CRDs (Equation (1)) [30]:

ΔG = 0.0038165 × AP_calRW − 0.22506 × MMGBSA dG Bind vdW − 0.24626 × HBOND2 − 0.049875 × FIREDOCK_AB − 3.3475(1)

This model utilises a series of protein‒protein docking scoring functions (AP_calRW, HBOND2, FIREDOCK_AB) to assess the protein‒protein contribution to the binding energy, as well as a function to consider the lipid‒protein contribution to the binding energy (MMGBSA dG Bind vdW). The model was built and validated against Wnt‒Fzd CRD binding affinity data derived by biolayer interferometry [31]. This model was selected as it afforded the most accurate predictions in both the training and test sets, as given by low root-mean-square errors (RMSE) between the predictions and experimental values, and by having a majority of the predictions occurring within the error range of the experiments. A peculiar feature of this model is that the coefficient of the lipid‒protein term in our model, which is derived from the Prime MM-GB/SA van der Waals component, is negative; since the values calculated by MMGBSA dG vdW are typically negative, the net result is that this term contributes positively—and hence, unfavourably—to the predicted binding energy. This is at odds with the majority of experimental findings that suggest the lipid is essential for Wnt signalling [4,32], although one recent study suggests that Wnt lipidation is not essential for Wnt signalling in specific contexts [33].

In this study, we have aimed to estimate the binding affinities for all human Wnts with all human proteins bearing putative Fzd CRDs. We applied our previously derived Wnt‒Fzd CRD binding affinity prediction model, as well as developed a new binding affinity prediction model, forcing the lipid‒protein contribution in the model to be favourable, thus better accounting for the role of Wnt lipidation in binding. While the new model affords an increased error compared to the previous model, the majority of binding affinities for Wnt‒Fzd and Wnt‒sFRP interactions are predicted to occur in similar ranges to the previous model. Applying the new model to estimate Wnt‒putative Fzd binding affinities implicates several of these in previously unexplored roles in effecting and modulating Wnt signalling. The results here will inform investigations of Wnt signalling modulation beyond Wnt‒Fzd and Wnt‒sFRP interactions.

## 2. Results

**Figure 1:**
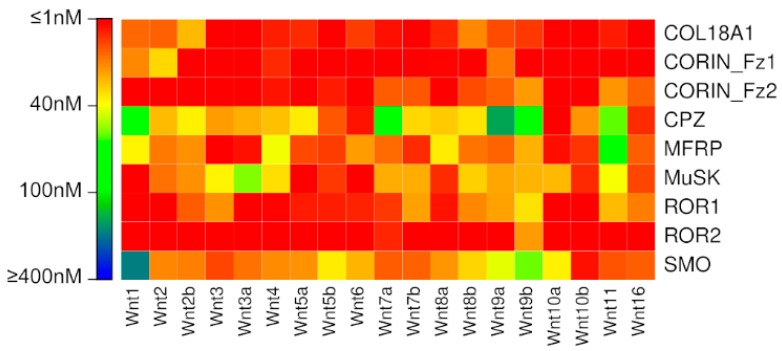
Binding affinities predicted for putative Fzd CRD interactions by the previously published model (Agostino, et al. 2017).

To our surprise, our previously published binding affinity prediction model suggests very strong binding affinities for nearly all Wnt interactions with the putative Fzd-type CRDs (Figure 1, Table S2). As the majority of these interactions have never been experimentally implicated in Wnt signalling, this seems an unlikely outcome, and prompted us to investigate alternative affinity prediction models.

**Figure 2:**
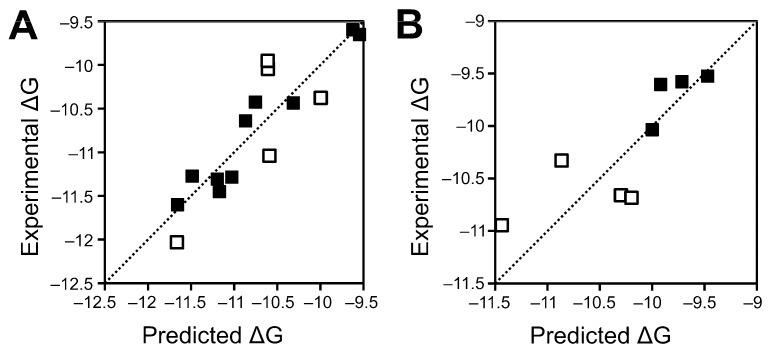
Comparison of experimental and predicted binding energies by Model 1 for training (**A**) and test (**B**) sets.

As noted in the introduction, lipidation is generally essential for mediating protein‒protein interactions involving Wnt proteins, thus expected to contribute favourably in Wnt‒protein binding energy, while in our previous model, this component will generally make an unfavourable contribution. Thus, we aimed to identify new models wherein the lipid‒protein term contributes favourably to the calculated binding energy. In repeating the model building and evaluation process and forcing this term to contribute favourably, we have identified several new Wnt‒Fzd CRD binding affinity prediction models (Table 1, Figure 2). As found previously [30], a four-descriptor model appears to be the optimal model for binding affinity prediction, with limited improvement observed when going to a five-descriptor model (data not shown). None of the obtained models provide quite as low an RMSE in either training or testing as compared to our previously published model; the best model, described in more detail in the next paragraph, yields binding energies within experimental error for at least half of the training and test sets, with an RMSE around 0.35 kcal/mol.

The best four-descriptor model features the residue-level interaction two-step potential described by Dror [34], as well as the E_local_ Cβ and E_local_ R_min_ statistical potential constituent terms described by Feliu et al. [35]; we have previously identified potentials related to both of these as being useful for pose prediction of Fc‒protein complexes [36]. The protein‒lipid term incorporated into the model is the MMGBSA dG Bind Solv GB term, which describes the energy associated with desolvating the protein binding site to facilitate ligand (in this case, lipid) binding. As this term is almost invariably positive (as desolvation is energetically unfavourable), the negative co-efficient against this term results in a negative value for the term, thus resulting in a net favourable contribution to the overall binding energy. Furthermore, while the previous model incorporates a number of atomic level potentials, the new model only incorporates residue-level potentials, which are likely to perform better with lower quality or predicted structures such as employed here [36]. Testing suggests that the new model is more likely to overestimate binding energies than the previous model, particularly for high-affinity interactions. Interactions between Wnt5 and Wnt5b appear to be particularly likely to be miscalculated, with the affinity of an equal number of complexes significantly over- and underestimated (Table 2).

**Figure 3:**
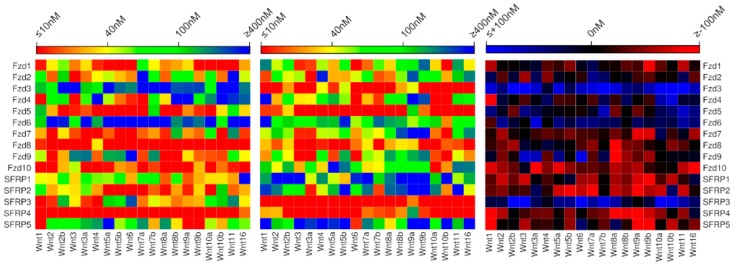
Comparison of binding affinities for all human Wnt‒Fzd and Wnt‒sFRP interactions predicted by the new model (**left**) and the previously published model (**center**), with differences between the models enumerated (**right**).

In comparing the energies calculated by the new model to those obtained by the previous model for the Wnt‒Fzd and Wnt‒sFRP interactions (Figure 3, Table S3), we find that 25% of these are predicted to be in the same affinity range by the two models, about 40% are in adjacent affinity ranges, and the remaining 35% predicted more than one affinity range apart. Particularly striking differences occur between the predictions made by the two models for SFRP1, SFRP2 and SFRP5, all of which are predicted by the new model to exhibit the same broad specificity as SFRP3 and SFRP4.

**Figure 4:**
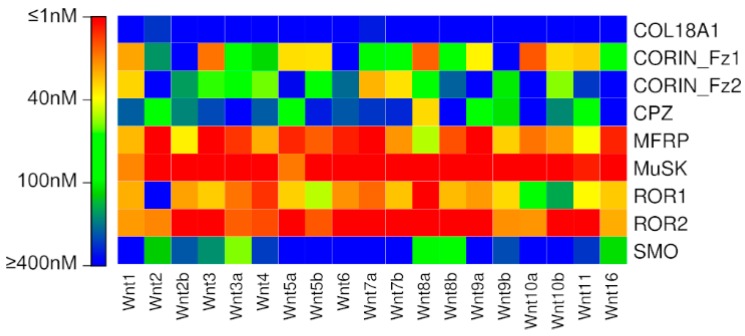
Binding affinities predicted for putative Fzd CRD interactions using the new model.

Several trends are noticeable in the predictions of Wnt interactions with putative Fzd CRDs obtained using the new model (Figure 4, Table S4). Firstly, all Wnts are predicted to bind to MuSK with high affinity. Almost all Wnts are predicted to bind to MFRP and ROR2 with high affinity. Approximately half of the Wnts are predicted to bind to ROR1 with high affinity. The two CORIN Fzd-type CRDs display high affinity for selected Wnts, and exhibit largely complementary specificities to one another; that is, Wnts bound with high affinity by the CORIN Fz1 domain are usually bound with low affinity by the CORIN Fz2 domain, and vice versa. Only Wnt8a is predicted to have high affinity for CPZ, and only Wnt3a is predicted to have at least moderate affinity for SMO. COL18A1 is not predicted to have high affinity for any Wnts.

## 3. Discussion

The striking difference in the Wnt‒Fzd and Wnt‒SFRP binding affinities predicted by the previous and the new model in selected cases illustrates the target-dependent nature of these types of interactions. Specifically, the CRDs for which the greatest deviation in Wnt affinity between the two models was observed come from two relatively well-conserved subfamilies of Fzd CRDs: SFRP1, SFRP2 and SFRP5 form a subgroup, while Fzd3 and Fzd6 form another subgroup. Further experimental knowledge of Wnt‒Fzd CRD binding affinities would be invaluable for generating more consistent affinity prediction models.

The prediction that many Wnts are capable of binding to ROR1 and ROR2 with high affinity suggests that numerous Wnts are capable of participating in non-canonical Wnt signalling. It is worth reiterating here that during training and testing, interactions with mWnt5 and mWnt5b proved to be some of the most likely to exhibit large variations. Similarly, MuSK is predicted to bind with high affinity to all Wnts. MuSK is also known to bind to Dishevelled; however, this has not been demonstrated in the context of Wnt signalling [26].

The prediction that the only Wnt capable of binding to SMO with at least moderate affinity is Wnt3a is quite interesting, considering that both of these proteins are implicated in numerous cancer types [37,38,39], albeit studied in distinct signalling contexts. SMO has never been reported to participate in Wnt signalling, but as the only protein other than the Fzds in the Class F GPCR family, it is not inconceivable that SMO could participate in Wnt signalling. Interestingly, it has been demonstrated that Wnt3a can activate Hedgehog signalling through the β-catenin/TCF4 mediated transcription of Hedgehog target genes, SMO, PTCH1 and GLI1, indicating considerable cross-talk between these two pathways [40]. Signalling through SMO is known to utilise G proteins, as is Wnt‒Ca2+ signalling [41], which could implicate SMO as a potential mediator of Wnt‒Ca2+ signalling. The lack of conservation between SMO and the Fzds of known Dvl-binding residues in the intracellular loops between SMO suggests that SMO is unlikely to utilise Dvl as an adapter protein to promote intracellular signalling (Table S5).

The complementary Wnt-binding specificities displayed by the two CORIN Fzd-type CRDs suggest that it may be able to bind to different Wnt ligands, depending on the physiological context. CORIN expression is enriched in heart muscle, although has also been reported to be highly expressed in the dermal papilla (DP) of hair follicles [42]. The activation of β-catenin signalling in the DP induces the expression of CORIN. Furthermore, the expression of Wnt ligands in the DP seems to be limited to Wnt5a [42], which our model predicted to bind with moderate affinity to CORIN‒Fz1. Our model also predicted a high binding affinity between Wnt1 and CORIN‒Fz1, which have been indirectly linked through the negative regulation of Wnt1-induced β-catenin accumulation in colorectal cancer cells by CORIN’s major substrate, ANP [19].

Although the Fzd CRD of COL18A1 has been demonstrated to modulate Wnt signalling by sequestering Wnt3a [22], no Wnts are predicted to bind to COL18A1 with high affinity; all Wnt‒COL18A1 interactions are predicted to occur in approximately the 0.2–2 μM range. While this is orders of magnitude lower than other Wnt‒Fzd interactions, it is well within the range of many physiologically relevant protein‒protein interactions [43]. Furthermore, COL18A1 strongly binds heparin, which in turn, could be potentiating the Wnt‒COL18A1 interaction; including carbohydrates—in particular, glycosaminoglycans—in structure and affinity prediction models is extremely challenging due to their high flexibility and hydrogen-bonding propensity [44,45,46].

Several recent studies suggest that Fzd-type CRDs may dimerise in the presence of free lipids, and that this dimerization is functionally significant [5,47]. CRD dimerization may be relevant in the context of the function of proteins with putative Fzd CRDs; in this case, the Fzd-type CRD may be acting to facilitate the lipid-mediated dimerization of the protein, rather than afford a function related to Wnt binding or Wnt signalling. In light of recent structural data for a mammalian Wnt‒Fzd interaction demonstrating CRD dimerization in the crystal packing [48], the affinity prediction models derived in this study and our previous work could be used to explore Wnt binding to CRD homo- and hetero-dimers. While specific kinetic data for Wnt‒CRD dimer interactions are not available at present, quantitative data derived from Fzd oligomerisation assays are available [49,50] and may assist with validating structural and functional predictions for Wnt interactions with Fzd CRD dimers.

In conclusion, we have predicted binding affinities for all human Wnts to all human putative Fzd CRDs using a newly validated affinity prediction model. The results of this study will guide the focus of future experimental investigations on specific Wnt‒Fzd interactions.

## 4. Materials and Methods

### 4.1. Homology Modelling, Complex Generation and Refinement

Homology models of the 19 human Wnt proteins have been previously prepared [30]. Homology models of the putative Fzd-type CRDs—with the exception of the SMO CRD, which was taken directly form the crystal structure complex of SMO with cholesterol (PDB 5L7D) [23]—were prepared against one of three templates, as detailed in Table 3: the structure of the rat MuSK CRD (PDB 3HKL) [51], the structure of the human Fzd7 CRD in complex with a C24 lipid (PDB 5URV) [5] and the structure of the mouse Fzd8 CRD in complex with Xenopus Wnt8 (PDB 4F0A) [52]. For structures modelled against the rat MuSK CRD, chain B in the structure was used; this chain was selected over chain A as a greater amount of the domain is resolved. For structures modelled against the human Fzd7 CRD complex with the C24 lipid, the lipid was retained during model building. For structures modelled against PDB 4F0A, the Wnt lipid was retained during model building. Sequence alignments generated for the homology modelling are provided in the Supplementary Materials (Table S1). Wnt complexes with putative Fzd CRDs were assembled and refined via minimisation and side-chain prediction using Prime (Schrodinger Suite, Schrodinger, New York NY, United States) as previously described [30], using a KNIME workflow to automate the process.

### 4.2. Prediction of Wnt Binding Affinities for Putative Fzd-Type CRDs

The refined complexes were analysed using CCharPPI [53], which contains over 100 scoring functions used for investigating protein‒protein interactions, and Prime MMGBSA. CCharPPI facilitated scoring of the refined complexes using the functions contained in our previously published model [30]: AP_calRW [54], HBOND2 [55] and FIREDOCK_AB [56]. Binding energies were then calculated according to this model. New affinity prediction models were generated wherein the lipid‒protein contribution to the binding energy was favourable. Utilizing the modelled complexes, scores and binding affinity data for the known mouse Wnt‒Fzd CRD complexes [31] we previously generated [30] (listed in Table 2), we repeated the model building and evaluation step using Strike (Schrodinger Suite) and an inhouse script, as previously described [30], with the additional condition that the lipid‒protein contribution to the binding energy must be favourable. Binding affinity plots were prepared using the ggplot2 module of R.

## Figures and Tables

**Table 1:** Best performing four-descriptor models predicting Wnt‒Fzd CRD binding energy obtained in this study ^a^.

Number	Model	RMSE_train_	RMSE_test_	InExp_train_	InExp_test_
1	ΔG = 0.06715 × CP_TSC + 0.001913 × CP_ELOCAL_CB − 0.01128 × CP_ELOCAL_MIN − 0.3072 × MMGBSA dG Bind Solv GB − 6.2941	0.33	0.36	9/15	4/8
2	ΔG = 0.002936 × CP_ELOCAL_CB − 0.01811 × CP_ELOCAL_MIN − 0.6022 × CP_ZLOCAL_CB − 0.2115 × MMGBSA dG Bind Solv GB − 7.2704	0.40	0.32	6/15	4/8
3	ΔG = 0.0840 × CP_TSC + 0.2258 × INSIDE − 0.06487 × FA_PP − 0.4274 × MMGBSA dG Bind Solv GB − 4.6833	0.36	0.40	7/15	6/8

^a^ ΔG and RMSE values in kcal/mol. InExp_train/test_ refers to the number of complexes for which ΔG was predicted within the error range reported by the experiment.

**Table 2:** Comparison of predictions made by Model 1 with experimental data for the training and test sets.

Interaction	ΔG_exp_^a^	ΔG_pred_^a^	|ΔG_exp_ − ΔG_pred_| ^a,b^	Experimental K_d_^c^	Predicted K_d_^c^	Experimental Range (Predicted Range) ^d^	Set
mWnt3a–mFzd2	−10.64	−10.87	0.23	15.7	10.7	+++ (+++)	Training
mWnt3a–mFzd4	−11.27	−11.49	0.22	5.4	3.7	++++ (++++)	Training
mWnt3a–mFzd5	−11.60	−11.66	0.06	3.1	2.8	++++ (++++)	Training
mWnt3a–mFzd7	−11.28	−11.03	0.25	5.3	8.1	++++ (++++)	Training
mWnt3a–mFzd8	−12.03	−11.66	0.37	1.5	2.8	++++ (++++)	Training
mWnt5–mFzd2	−10.38	−10.00	0.38	24.4	46.3	+++ (++)	Training
mWnt5–mFzd4	−10.38	−10.75	0.37	24.4	13.0	+++ (+++)	Training
mWnt5–mFzd5	−11.31	−11.20	0.11	5.1	6.1	++++ (++++)	Training
mWnt5–mFzd7	−10.05	−10.61	0.56	42.6	16.5	++ (+++)	Training
mWnt5–mFzd8	−11.45	−11.17	0.28	4.0	6.4	++++ (++++)	Training
mWnt5b–mFzd2	−9.60	−9.62	0.02	91.0	87.9	++ (++)	Training
mWnt5b–mFzd4	−9.95	−10.61	0.66	50.4	16.5	++ (+++)	Training
mWnt5b–mFzd5	−10.44	−10.32	0.12	22.0	27.0	+++ (+++)	Training
mWnt5b–mFzd7	−9.65	−9.55	0.10	83.7	99.0	++ (++)	Training
mWnt5b–mFzd8	−11.04	−10.59	0.45	8.0	17.1	++++ (+++)	Training
mWnt3a–mFzd1	−10.66	−10.30	0.36	15.2	27.9	+++ (+++)	Test
mWnt4–mFzd2	−9.53	−9.47	0.06	102.5	113.3	+ (+)	Test
mWnt4–mFzd4	−10.04	−10.00	0.04	43.3	46.3	++ (++)	Test
mWnt4–mFzd5	−10.68	−10.20	0.38	14.7	33.0	+++ (+++)	Test
mWnt4–mFzd7	−9.58	−9.72	0.14	94.2	74.3	++ (++)	Test
mWnt4–mFzd8	−10.95	−11.44	0.49	9.3	4.1	++++ (++++)	Test
mWnt5–mFzd1	−10.33	−10.87	0.44	26.5	10.7	+++ (+++)	Test
mWnt5b–mFzd1	−9.60	−9.92	0.32	91.0	53.0	++ (++)	Test

*^a^* Experimental ΔG (ΔG_exp_) calculated from experimental K_d_ values as ΔG = *RT* ln K_d_ where *R* is the gas constant (1.987 × 10^−3^ kcal K^−1^ mol^−1^) and *T* is the temperature at standard conditions (298 K). Predicted ΔG (ΔG_pred_) calculated according to Model 1. ΔG values expressed as kcal/mol. *^b^* Absolute value of difference between experimental and predicted ΔG values. *^c^* K_d_ values were obtained from Dijksterhuis et al. and represent the average values reported. All K_d_ values are expressed in nM. *^d^* Guide to affinity range classifications: <10 nM, ++++; 10–40 nM, +++; 40–100 nM, ++; 100–400 nM, +; >400 nM, −. Cases in which the experimental and predicted K_d_ values occur in different ranges are underlined. The range in which the value of experimental K_d_ occurs is shown outside parentheses; the range in which the predicted K_d_ value occurs is shown inside parentheses.

**Table 3:** Putative Fzd CRDs used in this study.

Protein	UniProt Accession	Sequence Used	Modelled Against ^a^
MFRP	Q9BY79	461–579	5URV
CPZ	Q66K79	27–160	5URV
CORIN (Fzd 1)	Q9Y5Q5	134–259	5URV
CORIN (Fzd 2)	Q9Y5Q5	450–573	4F0A
COL18A1	P39060	329–446	4F0A
SMO	Q99835	65–181	5L7D ^b^
MuSK	O15146	312–450	3HKL
ROR1	Q01973	165–299	3HKL
ROR2	Q01974	169–303	3HKL

^a^ Sequence alignments between sequences and templates listed in the Supplementary Materials (Table S1). ^b^ CRD extracted from this structure.

## Supplementary Materials

Supplementary materials can be found at https://www.mdpi.com/1422-0067/20/17/4168/s1. Table S1. Sequences alignments for homology modelling of putative Fzd domains. Table S2. Descriptor values, predicted binding energy and predicted dissociation constants for all Wnt‒putative Fzd CRD interactions using the previously published model. Table S3. Descriptor values, predicted binding energy and predicted dissociation constants for all human Wnt‒Fzd and Wnt‒SFRP interactions with the new model. Table S4. Descriptor values, predicted binding energy and predicted dissociation constants for all Wnt‒putative Fzd CRD interactions. Table S5. Aligned sequences of Class F GPCR intracellular loops (ICLs).

## Abbreviations

ANP	Atrial natriuretic peptide
APC	Adenomatous polyposis coli
COL18A1	Collagen XVIII α-chain
CORIN	Atrial natriuretic peptide-converting enzyme
CPZ	Carboxypeptidase Z
CRD	Cysteine-rich domain
DP	Dermal papilla
Dvl	Dishevelled
Fzd	Frizzled
GSK	Glycogen synthase kinase
LRP	Low-density lipoprotein receptor
MFRP	Membrane Frizzled-related protein
MMGBSA	Molecular mechanics generalized Born surface area
MuSK	Muscle skeletal receptor tyrosine-protein kinase
PCP	Planar cell polarity
PDB	Protein Data Bank
ROR	Receptor tyrosine kinase-like orphan receptor
sFRP	Secreted Frizzled-related protein
SMO	Smoothened
