# Supplementary material for: Wnt Binding Affinity Prediction for Putative Frizzled-Type Cysteine-Rich Domains
Source: Int J Mol Sci. 2019 Aug 26;20(17):4168. doi: 10.3390/ijms20174168 (PMC6747125; doi:10.3390/ijms20174168)
Supplement: Supplementary file 1 [file ijms-20-04168-s001.pdf]

Supporting Information

Table S1. Sequences alignments for homology modelling of cryptic Fzd domains.

MFRP	PPELACEPVQVEMCLGLSYNTTAFPNIWVGMITQEEVVEVLSGYKSLTSL
hFzd7 CRD	PDHGFCQPISIPLCTDIAYNQTILPNL-LGHTNQEDAGLEVHQFYPLVKV
	* *:*: : :* ::** * **: :* **: : : * :
MFRP	PCYQHFRRLLCGLLVPRCTPLGSVLPPCRSVCQEAEHQCQSGLALLGTPW
hFzd7 CRD	QCSPELRFFLCSMYAPVCTVLDQAIPPCRSLCERARQGCEALMNKFGFQW
	* * ** : * ** * :*****:*: * *:: : * *
MFRP	P--FNCNRLPEAADLEACAQP
hFzd7 CRD	PERLRCENFPVHGAGEICVGQ
	* * * * *
CPZ	NPAGECHRPPAADSATCVDLQLRTCSDAAYNHTTFPNLLQHRSWEVVEAS
hFzd7 CRD	QPYHGEKGISVPDHGFCQPISIPLCTDIAYNQTILPNLLGHTN-----QE
	* * * : : *:* *** * **** *
CPZ	SEYILLSVLHQLLEGQCNPDLRLLGCAVLAPRCEG-GWVRRPCRHICEGL
hFzd7 CRD	DAGLEVHQFYPLVKVQCSPELRFFLCSMYAPVCTVLDQAIPPCRSLCERA
	: : : *:: **:*:** *:: ** * *** :**
CPZ	REVCQPAFDAIDMAWPYFLDCHRYFTR-EDEGCYDP
hFzd7 CRD	RQGCEALMNKFGFQWPERLRCENFPVHGAGEICVGQ
	*: *: : ** * * : * *
CORIN Fzd1	RNTSACMNITHSQCQMLPYHATLTPLLSVVRNMEMEKFLK-FFTYLHRLS
hFzd7 CRD	PDHGFCQPISIPLCTDIAYNQTILPNLLGHTNQEDAGLEVHQFYPLVKVQ
	: * *: * : *: *: * * * * * * ::
CORIN Fzd1	CYQHIMLFGCTLAFPECIIDGDDSHGLLPCRSFCEAAKEGCESVLGMVNY
hFzd7 CRD	CSPELRFFLCSMYAPVCTVL---DQAIPPCRSLCERARQGCEALMNKFGF
	* : * *:: * * : : **** ** *::***::: *
CORIN Fzd1	SWPDFLRCSQFRNQTESSNVSRICFSP
hFzd7 CRD	QWPERLRCENFPVHGAG----EICVGQ
	**: *** * **
CORIN Fzd2	NNCSQCEPITLELCMNLPYNSTSYPNYFGHRTQKEASISWESSLFPALVQ
mFzd8 CRD	AKELACQEITVPLCKGIGYNYTYMPNQFNHDTQDEAGLEVH-QFWP-LVE
	*: **: ** : ** * ** * * ** ** : :* **:
CORIN Fzd2	TNCYKYLMFFSCTILVPKCDVNTGEHIPPCRALCEHSKERCESVLGIVGL
mFzd8 CRD	IQCSPDLKFFLCSMYTPICLEDYKKPLPPCRSVCERAKAGCAPLMRQYGF
	* * ** *:: * * : : :****::** :* * :: *
CORIN Fzd2	QWPEDTDCSQFPEENSDNQTCLMP
mFzd8 CRD	AWPDRMRCDRLPEQGNPDTLCMDA
	**: * : **: : : *:

COL18A1	APAGRCLPLPPSLPVCGHLGISRFWLPNHLHHESGEQVRAGARAWGGLLQ

```
mFzd8 CRD   ASAKELACQEITVPLCKGIGYNYTYMPNQFNHDTQDEAGLEVHQFWPLVE
            * *       ::*:*  :* :  ::**  :*:: ::      :  *::

COL18A1     THCHPFLAWFFCLLLVPPCGSVPPPAPPPCCQFCEALQDACWSRLG--GG
mFzd8 CRD   IQCSPDLKFFLCSMYTPICLEDYKKPLPPCRSVCERAKAGCAPLMRQYGF
             * * * :* * :  * *       ***   **  :  *   :   *

COL18A1     RLP--VACASLPTQ--EDGYCVLI
mFzd8 CRD   AWPDRMRCDRLPEQGNPDTLCMDA
             *  : *  ** *   *  *:
----------------------------------------------------------
MuSK        DNKGYCAQYRGEVCNAVLAKDALVFLNTSYADPEEAQELLVHTAWNELKV
rMuSK       GSKGYCAQYRGEVCDAVLVKDSLVFFNTSYPDPEEAQELLIHTAWNELKA
             :************:*** **:*** **** *********:********

MuSK        VSPVCRPAAEALLCNHIFQECSPGVVPTPIPICREYCLAVKELFCAKEWL
rMuSK       VSPLCRPAAEALLCNHLFQECSPGVLPTPMPICREYCLAVKELFCAKEWL
            ***:************:********:***:********************

MuSK        VMEEKTHRGLYRSEMHLLSVPECSKLPSMHWDPTACARL
rMuSK       AMEGKTHRGLYRSGMHFLPVPECSKLPSMHQDPTACTRL
             ** ********* ** * *********** ***** **
----------------------------------------------------------
ROR1        EEDGFCQPYRGIACARFIGNRTVYMESLHMQGEIENQITAAFTMIGTSSH
rMuSK       GSKGYCAQYRGEVCDAVLVKDSLVFFNTSYPDPEEAQELLIHTAWNELKA
               *:*  ***  *   :  ::          * *     *

ROR1        LSDKCSQFAIPSLCHYAFPYCDETSSVPKPRDLCRDECEILENVLCQTEY
rMuSK       VSPLCRPAAEALLCNHLFQECSP-GVLPTPMPICREYCLAVKELFCAKEW
            :*  *   *   **:: *  *     :* *  :**: *  :: : *  *:

ROR1        IFARSNPMILMR------LKLPNCEDLP-QPESPEAANCIRI
rMuSK       LAMEGKTHRGLYRSGMHFLPVPECSKLPSMHQ--DPTACTRL
            :        :       * :* *  **   :  :   * *:
----------------------------------------------------------
ROR2        HEDGFCQPYRGIACARFIGNRTIYVDSLQMQGEIENRITAAFTMIGTSTH
rMuSK       GSKGYCAQYRGEVCDAVLVKDSLVFFNTSYPDPEEAQELLIHTAWNELKA
               *:*  ***  *   :  ::  :      * :     *

ROR2        LSDQCSQFAIPSFCHFVFPLCDARSRTPKPRELCRDECEVLESDLCRQEY
rMuSK       VSPLCRPAAEALLCNHLFQECSP GVLPTPMPICREYCLAVKELFCAKEW
            :*  *   *    *: :* *       * *  :**: *  ::   * :*:

ROR2        TIARSNPLILMR------LQLPKCEALP-MPESPDAANCMRI
rMuSK       LAMEGKTHRGLYRSGMHFLPVPECSKLPSMHQ--DPTACTRL
                     :       * :*:*  ** * :  *   * *:
----------------------------------------------------------
```

Table S2. Descriptor values, predicted binding energy and predicted dissociation constants for all Wnt-cryptic Fzd CRD interactions using the previously published model.

PROTEINS		DESCRIPTORS				BINDING AFFINITY		
Wnt	CRD	AP_calRW	MMGBSA dG Bind vdW	HBOND2	FIREDOCK_AB	ΔG	K_d	Approx Strength
Wnt1	COL18A1	-5117.01	-26.25	-3.23	-95.28	-11.42	4.20	++++
	CORIN Fz1	-5592.29	-30.93	-4.48	-109.21	-11.18	6.31	++++
	CORIN Fz2	-5200.69	-28.34	-3.78	-65.88	-12.60	0.57	++++
	CPZ	-4520.88	-31.69	-3.05	-57.63	-9.84	60.48	++
	MFRP	-4711.10	-29.94	-1.79	-77.06	-10.30	27.72	+++
	MuSK	-5361.26	-30.50	-1.51	-70.04	-13.08	0.26	++++
	ROR1	-5230.64	-33.61	-6.67	-73.41	-10.44	21.93	+++
	ROR2	-5284.74	-21.47	-5.00	-80.41	-13.44	0.14	++++
	SMO	-4172.37	-26.92	-3.92	-62.47	-9.13	201.12	+
Wnt2	COL18A1	-4933.90	-25.45	-4.47	-77.56	-11.48	3.81	++++
	CORIN Fz1	-5907.88	-35.35	-6.63	-115.37	-10.55	18.23	+++
	CORIN Fz2	-5537.40	-26.91	-5.22	-85.69	-12.87	0.37	++++
	CPZ	-4916.70	-33.60	-2.52	-63.75	-10.75	13.02	+++
	MFRP	-4854.69	-28.28	-1.99	-74.33	-11.31	5.03	++++
	MuSK	-5344.26	-33.35	-3.31	-81.32	-11.37	4.62	++++
	ROR1	-5396.44	-26.74	-6.60	-105.92	-11.02	8.33	++++
	ROR2	-5551.93	-23.94	-3.39	-81.77	-14.24	0.04	++++
	SMO	-4435.06	-22.99	-2.38	-66.72	-11.19	6.26	++++
Wnt2b	COL18A1	-4700.98	-26.20	-4.83	-68.32	-10.80	12.08	+++
	CORIN Fz1	-5644.51	-29.74	-2.86	-101.77	-12.42	0.78	++++
	CORIN Fz2	-5443.57	-28.63	-4.19	-88.55	-12.23	1.07	++++
	CPZ	-4648.68	-31.97	-2.17	-60.18	-10.36	25.26	+++
	MFRP	-4644.01	-27.61	-2.12	-64.53	-11.12	7.02	++++
	MuSK	-5146.47	-32.57	-2.72	-77.6	-11.12	7.03	++++
	ROR1	-5297.51	-28.17	-6.36	-92.27	-11.06	7.76	++++
	ROR2	-4999.85	-23.75	-4.36	-66.61	-12.69	0.49	++++
	SMO	-4345.68	-23.74	-2.16	-56.88	-11.22	5.90	++++
Wnt3	COL18A1	-5373.49	-25.07	-2.55	-102.56	-12.47	0.71	++++
	CORIN Fz1	-6296.73	-32.31	-4.50	-123.21	-12.85	0.37	++++
	CORIN Fz2	-5668.24	-27.44	-5.89	-89.95	-12.87	0.36	++++
	CPZ	-4817.98	-30.67	-2.44	-64.41	-11.02	8.28	++++
	MFRP	-5214.13	-26.07	-2.46	-81.62	-12.70	0.48	++++
	MuSK	-5396.82	-37.27	-2.64	-92.24	-10.31	27.62	+++
	ROR1	-5507.14	-23.84	-5.81	-92.29	-12.97	0.31	++++
	ROR2	-5227.51	-21.34	-4.39	-65.93	-14.13	0.04	++++
	SMO	-4638.59	-22.12	-4.31	-66.64	-11.69	2.68	++++
Wnt3a	COL18A1	-5387.81	-25.70	-1.58	-102.38	-12.63	0.54	++++
	CORIN Fz1	-6079.44	-30.47	-2.06	-119.22	-13.24	0.20	++++
	CORIN Fz2	-5584.65	-27.23	-4.38	-89.21	-13.01	0.29	++++
	CPZ	-4639.83	-31.99	-1.05	-54.37	-10.88	10.40	+++
	MFRP	-5123.45	-26.23	-1.44	-92.38	-12.04	1.49	++++
	MuSK	-5482.28	-35.32	-6.57	-93.53	-10.04	43.36	++
	ROR1	-5422.33	-32.14	-4.55	-93	-11.05	7.90	++++
	ROR2	-5391.88	-23.35	-3.95	-79.1	-13.75	0.08	++++

	SMO	-4646.92	-24.77	-3.31	-66.62	-11.37	4.58	++++
Wnt4	COL18A1	-5149.79	-26.10	-2.70	-90.27	-11.96	1.69	++++
	CORIN Fz1	-5818.32	-35.57	-2.51	-101.32	-11.88	1.95	++++
	CORIN Fz2	-5481.27	-28.24	-4.92	-93.81	-12.02	1.53	++++
	CPZ	-4675.16	-32.40	-2.14	-52.85	-10.74	13.38	+++
	MFRP	-4692.85	-28.50	-3.65	-74.49	-10.23	31.43	+++
	MuSK	-5328.82	-34.75	-3.20	-91.95	-10.49	20.29	+++
	ROR1	-5611.66	-28.41	-5.06	-97.37	-12.27	1.01	++++
	ROR2	-5038.73	-18.92	-5.99	-53.34	-14.19	0.04	++++
	SMO	-4510.69	-24.19	-3.33	-63.05	-11.15	6.61	++++
Wnt5a	COL18A1	-5118.84	-25.01	-3.16	-92.16	-11.88	1.94	++++
	CORIN Fz1	-5813.78	-30.18	-2.44	-99.59	-13.17	0.22	++++
	CORIN Fz2	-5347.28	-27.24	-5.41	-74.36	-12.58	0.59	++++
	CPZ	-4621.65	-32.57	-1.70	-57.41	-10.37	24.63	+++
	MFRP	-5099.39	-27.32	-3.76	-81.59	-11.67	2.77	++++
	MuSK	-5310.82	-28.56	-4.02	-77.84	-12.32	0.93	++++
	ROR1	-5403.93	-22.46	-7.61	-101.14	-12.00	1.59	++++
	ROR2	-5093.87	-21.77	-5.86	-44.31	-14.24	0.04	++++
	SMO	-4430.42	-20.85	-5.21	-63.94	-11.09	7.35	++++
Wnt5b	COL18A1	-5263.26	-24.94	-1.97	-90.31	-12.83	0.39	++++
	CORIN Fz1	-6158.33	-34.35	-4.52	-111.33	-12.46	0.73	++++
	CORIN Fz2	-5575.93	-29.20	-5.54	-94.46	-11.98	1.63	++++
	CPZ	-4774.91	-32.40	-4.10	-34.46	-11.55	3.39	++++
	MFRP	-5141.33	-27.11	-3.57	-85.18	-11.74	2.45	++++
	MuSK	-5405.70	-31.64	-4.08	-81.98	-11.76	2.35	++++
	ROR1	-5402.83	-30.14	-4.91	-66.3	-12.67	0.51	++++
	ROR2	-5261.29	-22.33	-4.37	-67.68	-13.95	0.06	++++
	SMO	-4545.06	-26.97	-2.35	-73.29	-10.39	23.97	+++
Wnt6	COL18A1	-5134.03	-25.70	-2.52	-96.25	-11.74	2.47	++++
	CORIN Fz1	-6033.47	-29.17	-2.29	-121.72	-13.18	0.22	++++
	CORIN Fz2	-5215.04	-27.22	-3.92	-75.93	-12.37	0.84	++++
	CPZ	-4801.98	-31.35	-1.75	-43.53	-12.02	1.54	++++
	MFRP	-4741.94	-27.57	-2.31	-73.56	-11.00	8.53	++++
	MuSK	-5616.10	-34.13	-2.71	-67.32	-13.08	0.26	++++
	ROR1	-5319.00	-28.63	-3.98	-72.16	-12.63	0.55	++++
	ROR2	-5278.98	-22.33	-4.29	-78.75	-13.49	0.13	++++
	SMO	-4381.51	-24.59	-3.26	-58.66	-10.81	11.87	+++
Wnt7a	COL18A1	-5099.09	-25.84	-3.00	-84.69	-12.03	1.50	++++
	CORIN Fz1	-5913.52	-29.44	-3.15	-98	-13.63	0.10	++++
	CORIN Fz2	-5442.43	-28.52	-5.70	-95.87	-11.52	3.58	++++
	CPZ	-4676.16	-34.08	-2.90	-63.9	-9.62	87.40	++
	MFRP	-4811.68	-26.96	-1.40	-78.16	-11.40	4.36	++++
	MuSK	-5238.32	-33.92	-3.93	-76.8	-10.91	9.99	++++
	ROR1	-5151.05	-26.23	-4.67	-71.68	-12.38	0.83	++++
	ROR2	-5149.61	-21.26	-5.02	-62.89	-13.84	0.07	++++
	SMO	-4579.50	-22.55	-4.66	-61.71	-11.52	3.53	++++
Wnt7b	COL18A1	-5292.79	-22.84	-3.03	-97.89	-12.78	0.42	++++
	CORIN Fz1	-5708.16	-32.01	-1.51	-101.61	-12.49	0.69	++++
	CORIN Fz2	-5662.73	-28.73	-6.98	-104.97	-11.54	3.44	++++
	CPZ	-4603.30	-30.16	-1.90	-62.67	-10.53	18.79	+++
	MFRP	-4874.81	-27.06	-1.81	-71.4	-11.86	2.01	++++

	MuSK	-5209.69	-33.63	-2.58	-82.63	-10.90	10.06	+++
	ROR1	-4975.09	-21.35	-4.89	-72.47	-12.71	0.48	++++
	ROR2	-5045.96	-22.96	-5.88	-79.67	-12.02	1.54	++++
	SMO	-4484.79	-24.25	-2.38	-59.01	-11.48	3.83	++++
Wnt8a	COL18A1	-5017.10	-25.18	-2.14	-88.4	-11.89	1.90	++++
	CORIN Fz1	-5529.75	-29.38	-3.47	-96.86	-12.15	1.22	++++
	CORIN Fz2	-5060.09	-27.03	-2.98	-62.94	-12.70	0.48	++++
	CPZ	-4496.56	-31.38	-1.68	-47.75	-10.65	15.44	+++
	MFRP	-4581.21	-30.43	-2.08	-62.08	-10.37	24.59	+++
	MuSK	-5188.16	-29.37	-3.49	-76.89	-11.84	2.06	++++
	ROR1	-5290.19	-26.71	-4.87	-92.58	-11.71	2.58	++++
	ROR2	-5168.31	-21.55	-4.87	-71.05	-13.48	0.13	++++
	SMO	-4307.05	-22.20	-3.51	-57.43	-11.06	7.74	++++
Wnt8b	COL18A1	-4817.24	-24.24	-4.57	-79.65	-11.18	6.31	++++
	CORIN Fz1	-6076.35	-31.08	-4.28	-123.72	-12.32	0.92	++++
	CORIN Fz2	-5174.83	-27.18	-5.71	-79.21	-11.62	2.99	++++
	CPZ	-4545.10	-31.95	-1.99	-51.49	-10.44	21.87	+++
	MFRP	-4703.84	-27.57	-0.89	-70.9	-11.34	4.83	++++
	MuSK	-5061.69	-35.13	-2.80	-69.5	-10.60	16.72	+++
	ROR1	-5428.04	-28.61	-5.64	-102.24	-11.14	6.80	++++
	ROR2	-5015.62	-21.85	-7.88	-75.07	-11.89	1.91	++++
	SMO	-4331.76	-25.04	-3.60	-55.69	-10.58	17.41	+++
Wnt9a	COL18A1	-5106.59	-26.14	-2.78	-92.75	-11.64	2.89	++++
	CORIN Fz1	-5783.75	-31.44	-3.83	-121.93	-11.32	4.99	++++
	CORIN Fz2	-5221.82	-27.25	-4.46	-91.87	-11.46	3.90	++++
	CPZ	-4723.03	-34.40	-3.42	-70.89	-9.26	162.98	+
	MFRP	-5035.54	-28.13	-3.05	-81.06	-11.44	4.07	++++
	MuSK	-5176.79	-33.37	-3.17	-77.93	-10.93	9.67	++++
	ROR1	-5396.60	-27.94	-4.48	-99.14	-11.61	3.08	++++
	ROR2	-5099.67	-22.61	-8.73	-75.37	-11.81	2.17	++++
	SMO	-4494.88	-26.29	-4.98	-63.4	-10.20	33.18	+++
Wnt9b	COL18A1	-5074.59	-26.26	-1.56	-93.5	-11.76	2.38	++++
	CORIN Fz1	-5841.41	-30.04	-1.36	-113.21	-12.90	0.35	++++
	CORIN Fz2	-4912.44	-26.25	-5.69	-75.19	-11.04	8.06	++++
	CPZ	-4673.90	-33.55	-1.85	-68.79	-9.75	70.81	++
	MFRP	-5066.59	-27.15	-2.76	-101.16	-10.85	11.05	+++
	MuSK	-5077.57	-31.89	-2.62	-81.94	-10.82	11.66	+++
	ROR1	-5360.07	-27.48	-4.85	-104.28	-11.22	5.87	++++
	ROR2	-5126.60	-22.02	-7.08	-75.85	-12.43	0.76	++++
	SMO	-4442.52	-23.79	-4.07	-78.88	-10.01	45.29	++
Wnt10a	COL18A1	-5246.57	-26.16	-2.32	-94.97	-12.18	1.18	++++
	CORIN Fz1	-5850.96	-31.41	-3.27	-105.25	-12.55	0.62	++++
	CORIN Fz2	-5694.32	-26.47	-5.18	-85.42	-13.59	0.11	++++
	CPZ	-4805.75	-28.58	-1.84	-50.69	-12.28	0.99	++++
	MFRP	-4818.78	-26.77	-2.53	-60.65	-12.07	1.42	++++
	MuSK	-5367.47	-32.62	-5.52	-87.04	-10.79	12.15	+++
	ROR1	-5223.03	-24.46	-5.12	-78.43	-12.61	0.57	++++
	ROR2	-5313.07	-23.05	-5.02	-75.57	-13.43	0.14	++++
	SMO	-4406.27	-24.67	-4.84	-61.35	-10.36	25.26	+++
Wnt10b	COL18A1	-5195.21	-25.99	-2.48	-89.39	-12.26	1.02	++++
	CORIN Fz1	-5856.04	-31.79	-2.49	-99.71	-12.96	0.31	++++

	CORIN Fz2	-5329.57	-27.16	-4.16	-73.08	-12.91	0.34	++++
	CPZ	-4957.70	-31.09	-3.72	-65.88	-11.07	7.59	++++
	MFRP	-4647.59	-26.07	-2.54	-56.22	-11.79	2.26	++++
	MuSK	-5446.21	-32.37	-3.84	-80.67	-11.88	1.94	++++
	ROR1	-5364.37	-25.97	-4.83	-80.73	-12.76	0.44	++++
	ROR2	-5324.25	-22.83	-3.73	-70.23	-14.11	0.04	++++
	SMO	-4516.56	-26.01	-3.80	-35.31	-12.03	1.49	++++
Wnt11	COL18A1	-5317.26	-25.71	-2.39	-105.81	-11.99	1.61	++++
	CORIN Fz1	-5833.68	-30.14	-2.21	-107.37	-12.93	0.33	++++
	CORIN Fz2	-5446.99	-27.98	-6.35	-104.41	-11.07	7.63	++++
	CPZ	-4693.62	-33.72	-2.80	-59.8	-10.00	46.33	++
	MFRP	-4732.93	-30.91	-4.26	-70.67	-9.88	56.65	++
	MuSK	-5507.03	-34.80	-4.31	-104.9	-10.24	30.89	+++
	ROR1	-5244.76	-29.55	-3.33	-78.29	-11.99	1.61	++++
	ROR2	-5100.65	-23.23	-3.94	-71.09	-13.07	0.26	++++
	SMO	-4605.37	-25.51	-4.99	-47.61	-11.58	3.23	++++
Wnt16	COL18A1	-5614.11	-25.83	-1.37	-102.21	-13.53	0.12	++++
	CORIN Fz1	-6063.68	-32.76	-3.57	-117.82	-12.36	0.86	++++
	CORIN Fz2	-5881.53	-28.02	-10.10	-110.88	-11.47	3.86	++++
	CPZ	-5026.57	-30.64	-2.70	-62.39	-11.86	2.00	++++
	MFRP	-5102.34	-27.09	-2.71	-91.43	-11.50	3.70	++++
	MuSK	-5510.80	-33.65	-3.63	-84.85	-11.68	2.71	++++
	ROR1	-5550.58	-25.80	-6.43	-108.04	-11.75	2.40	++++
	ROR2	-5463.94	-22.02	-7.42	-78.98	-13.48	0.13	++++
	SMO	-4822.07	-22.93	-5.58	-74.77	-11.49	3.76	++++

[a]ΔG is in kcal/mol and calculated according to previously published model: ΔG = 0.0038165 × AP_calRW – 0.22506 × MMGBSA dG Bind vdW – 0.24626 × HBOND2 – 0.049875 × FIREDOCK_AB – 3.3475. K_d is in nM and calculated according to the following equation: $K_d = e^{\frac{\Delta G}{RT}} \times 10^9$ where ΔG is the binding energy predicted by the model, R is the gas constant (1.987×10^{-3} kcal/(K mol)) and T is standard ambient temperature (298K). Approximate strength based on ranges defined by Dijksterhuis et al. (1): ++++, <10nM; +++, 10-40nM; ++, 40-100nM; +, 100-400nM; -, >400nM.

Table S3. Descriptor values, predicted binding energy and predicted dissociation constants for all human Wnt-Fzd and Wnt-SFRP interactions with the new model.

PROTEINS		DESCRIPTORS				BINDING AFFINITY		
Wnt	CRD	CP_TSC	CP_ELOCAL_CB	CP_ELOCAL_MIN	MMGBSA dG Bind Solv GB	ΔG	K_d	Approx Strength
Wnt1	Fzd1	-16.58	-3494	-600.1	11.38	-10.82	11.65	+++
	Fzd2	-7.86	-3217	-563.5	10.38	-9.81	64.01	++
	Fzd3	-16.36	-2942	-520.9	7.21	-9.36	137.01	+
	Fzd4	-22.31	-3328	-601.8	12.78	-11.30	5.19	++++
	Fzd5	-13.42	-3176	-640.7	10.77	-9.35	138.45	+
	Fzd6	-17.36	-2925	-499.1	6.54	-9.43	120.64	+
	Fzd7	-18.37	-3338	-596	10.26	-10.34	26.04	+++
	Fzd8	-15.76	-3192	-564.5	11.95	-10.76	12.82	+++
	Fzd9	0.34	-3127	-548.3	12.73	-9.98	48.08	++
	Fzd10	-16.75	-3218	-569.9	14.15	-11.49	3.73	++++
	SFRP1	-11.92	-2542	-405	8.71	-10.06	41.53	++
	SFRP2	-16.35	-2901	-468.8	7.04	-9.82	63.12	++
	SFRP3	-14.02	-2790	-428.8	10.64	-11.00	8.53	++++
	SFRP4	-16.2	-3134	-504	16.85	-12.87	0.37	++++
	SFRP5	-31.44	-2821	-520.2	10.04	-11.02	8.32	++++
Wnt2	Fzd1	-7.72	-3504	-596.5	10.84	-10.11	38.17	+++
	Fzd2	-14.22	-3210	-523.2	10.13	-10.60	16.83	+++
	Fzd3	-24.1	-2795	-529.3	7.75	-9.67	81.20	++
	Fzd4	-9.3	-3692	-637.5	9.35	-9.66	82.18	++
	Fzd5	-17.27	-3385	-627.3	11.27	-10.31	27.30	+++
	Fzd6	3.91	-2772	-433	5.43	-8.12	1114.31	-
	Fzd7	-26.02	-3460	-604.6	10.20	-10.97	8.97	++++
	Fzd8	-18.53	-3113	-504.5	12.56	-11.66	2.81	++++
	Fzd9	-14.53	-3638	-622.5	11.93	-10.87	10.62	+++
	Fzd10	-13.44	-3362	-520.4	12.24	-11.52	3.57	++++
	SFRP1	-8.05	-2716	-403.9	8.63	-10.12	37.58	+++
	SFRP2	-14.13	-3033	-517.2	10.20	-10.34	25.97	+++
	SFRP3	-0.59	-3082	-447.1	11.61	-10.75	13.04	+++
	SFRP4	-12.88	-3291	-586.9	16.67	-11.95	1.71	++++
	SFRP5	-10	-2999	-548.5	9.84	-9.54	101.38	+
Wnt2b	Fzd1	-8.84	-3093	-539.7	10.41	-9.91	53.54	++
	Fzd2	-7.83	-3292	-590.4	9.75	-9.45	117.08	+
	Fzd3	-4.29	-2906	-561	6.28	-7.74	2096.88	-
	Fzd4	0.48	-3092	-574.6	12.46	-9.52	103.80	+
	Fzd5	-9.88	-3130	-469	11.45	-11.17	6.40	++++
	Fzd6	-11.38	-2949	-512.2	6.20	-8.83	336.82	+
	Fzd7	-22.31	-3048	-580.9	10.04	-10.15	35.76	+++
	Fzd8	-18.62	-3251	-545.1	13.32	-11.71	2.60	++++
	Fzd9	-9.96	-3401	-572.3	10.80	-10.33	26.53	+++
	Fzd10	-6.63	-3543	-603	11.68	-10.30	27.83	+++
	SFRP1	-13.44	-2627	-454.9	8.30	-9.64	84.99	++
	SFRP2	-13.62	-3234	-564.3	9.89	-10.07	41.40	++
	SFRP3	5.43	-3126	-525.6	10.97	-9.35	138.86	+
	SFRP4	-8.88	-3122	-524	16.05	-11.88	1.93	++++

	SFRP5	-11.01	-3151	-504.3	7.73	-9.75	71.00	++
Wnt3	Fzd1	-14.37	-3159	-554.9	11.26	-10.50	19.90	+++
	Fzd2	-23.06	-3375	-560.7	11.26	-11.43	4.13	++++
	Fzd3	-9.05	-2875	-510.5	6.38	-8.60	490.93	-
	Fzd4	-18.7	-3125	-526.5	10.67	-10.87	10.73	+++
	Fzd5	-20.57	-3416	-580.9	10.23	-10.80	12.04	+++
	Fzd6	-8.12	-3136	-532.4	9.61	-9.78	66.61	++
	Fzd7	-17.15	-3088	-521.2	10.38	-10.66	15.14	+++
	Fzd8	-20.23	-3334	-562.9	11.77	-11.30	5.18	++++
	Fzd9	1.15	-3612	-635.1	10.33	-9.13	200.07	+
	Fzd10	-1.11	-3662	-647.6	12.74	-9.98	47.85	++
	SFRP1	-13.62	-2904	-495.3	9.88	-10.21	32.39	+++
	SFRP2	-19.53	-2863	-500.5	8.91	-10.17	34.59	+++
	SFRP3	-4.73	-3481	-575.1	10.91	-10.13	36.99	+++
	SFRP4	-19.65	-2865	-505.5	16.99	-12.61	0.56	++++
	SFRP5	-16.08	-2917	-499.2	8.13	-9.82	62.81	++
Wnt3a	Fzd1	-12.96	-3176	-573.9	11.11	-10.18	34.34	+++
	Fzd2	-16.59	-2957	-488	9.71	-10.54	18.52	+++
	Fzd3	-13.74	-2828	-474.2	6.94	-9.41	125.81	+
	Fzd4	0.37	-3339	-571.6	11.90	-9.86	58.40	++
	Fzd5	-18.19	-3406	-613.1	11.51	-10.65	15.44	+++
	Fzd6	9.33	-3035	-522.1	6.15	-7.47	3312.71	-
	Fzd7	-26.58	-3530	-604.7	10.93	-11.37	4.60	++++
	Fzd8	-17.44	-3270	-548.2	11.69	-11.13	6.91	++++
	Fzd9	1.24	-3469	-610	10.21	-9.10	210.92	+
	Fzd10	-24.64	-3186	-536.5	13.22	-12.05	1.45	++++
	SFRP1	-17.42	-2902	-559.8	10.21	-9.84	61.05	++
	SFRP2	-15.84	-2690	-457.1	8.22	-9.87	57.41	++
	SFRP3	-5.17	-3247	-495.9	11.34	-10.74	13.22	+++
	SFRP4	-20.67	-3106	-541.1	16.31	-12.53	0.65	++++
	SFRP5	-14.04	-2865	-479.3	6.58	-9.33	143.04	+
Wnt4	Fzd1	-13.35	-3226	-514.5	10.42	-10.76	12.89	+++
	Fzd2	-20.06	-3677	-687.4	10.34	-10.10	39.38	+++
	Fzd3	-15.63	-3413	-581	7.16	-9.52	104.63	+
	Fzd4	7.75	-3451	-608.6	10.53	-8.74	386.89	+
	Fzd5	-15.96	-3521	-585	11.72	-11.10	7.20	++++
	Fzd6	-1.34	-3149	-552.8	10.11	-9.28	157.19	+
	Fzd7	-24.95	-3250	-593.7	11.02	-10.87	10.60	+++
	Fzd8	-7.98	-3633	-681	13.16	-10.14	36.62	+++
	Fzd9	-11.52	-3445	-640.2	13.51	-10.59	17.22	+++
	Fzd10	-18.98	-3372	-575.6	12.66	-11.41	4.25	++++
	SFRP1	-9.58	-2790	-454.8	9.00	-9.91	54.21	++
	SFRP2	-12.49	-2747	-441.1	9.69	-10.39	24.08	+++
	SFRP3	-13.11	-3428	-575.2	12.21	-10.99	8.66	++++
	SFRP4	-13.89	-3499	-573.1	15.94	-12.35	0.87	++++
	SFRP5	-14.69	-3257	-580.6	7.40	-9.23	168.81	+
Wnt5a	Fzd1	-13.14	-2817	-471.8	11.79	-10.87	10.73	+++
	Fzd2	-9.48	-3355	-584	10.53	-10.00	46.66	++
	Fzd3	-20.21	-3300	-648	5.77	-8.43	660.80	-
	Fzd4	-1.02	-3258	-545.8	11.49	-9.97	48.93	++
	Fzd5	-22.94	-3752	-676.3	11.80	-11.01	8.46	++++

	Fzd6	7.89	-3150	-600.3	9.74	-8.01	1330.89	-
	Fzd7	-10.11	-3306	-525.4	10.54	-10.61	16.58	+++
	Fzd8	-13.11	-3068	-519	12.96	-11.17	6.42	++++
	Fzd9	-1.37	-3375	-603.4	10.57	-9.28	155.53	+
	Fzd10	-14.98	-3550	-635.3	12.72	-10.83	11.36	+++
	SFRP1	-10.49	-2803	-514.2	10.99	-9.94	51.63	++
	SFRP2	-24.73	-2565	-462.8	11.83	-11.28	5.38	++++
	SFRP3	-9.85	-3348	-629.7	10.35	-9.44	119.93	+
	SFRP4	-7.21	-3111	-540.8	16.29	-11.63	2.94	++++
	SFRP5	-10.75	-2810	-489.2	6.02	-8.72	400.50	-
Wnt5b	Fzd1	-14.8	-2914	-467	11.17	-11.02	8.21	++++
	Fzd2	-14.65	-3353	-607.3	11.17	-10.27	29.21	+++
	Fzd3	-18.23	-2933	-493.2	4.89	-9.07	224.23	+
	Fzd4	-13.7	-3413	-591.6	11.26	-10.53	18.95	+++
	Fzd5	-20.06	-3196	-534.4	11.64	-11.30	5.13	++++
	Fzd6	-1.46	-3168	-631.6	9.37	-8.21	957.95	-
	Fzd7	-17.63	-3326	-538.3	10.10	-10.87	10.67	+++
	Fzd8	-18.91	-3051	-553.4	12.89	-11.12	7.01	++++
	Fzd9	-6.48	-3213	-534	7.85	-9.26	161.19	+
	Fzd10	-11.89	-3523	-681.4	13.84	-10.40	23.66	+++
	SFRP1	-12.58	-2639	-401.2	6.80	-9.75	70.65	++
	SFRP2	-27.9	-2980	-500.4	10.40	-11.42	4.22	++++
	SFRP3	-1.04	-2990	-505.6	10.16	-9.50	107.55	+
	SFRP4	-18.78	-3072	-507.6	17.03	-12.94	0.32	++++
	SFRP5	-8.83	-2775	-462.6	9.66	-9.94	50.84	++
Wnt6	Fzd1	-12.27	-3199	-499.5	11.27	-11.06	7.69	++++
	Fzd2	-5.34	-3451	-554	11.20	-10.45	21.81	+++
	Fzd3	-11.29	-2978	-531.8	7.85	-9.16	190.97	+
	Fzd4	-16.36	-3077	-517.1	10.83	-10.77	12.59	+++
	Fzd5	-24.51	-3575	-571	12.03	-12.03	1.50	++++
	Fzd6	-8.26	-3131	-525.2	5.65	-8.65	454.48	-
	Fzd7	-20.67	-3353	-564.2	10.76	-11.04	8.05	++++
	Fzd8	-19.42	-3471	-596.7	12.15	-11.24	5.71	++++
	Fzd9	2.2	-3636	-701	10.40	-8.39	703.61	-
	Fzd10	-7.63	-3482	-623.9	13.28	-10.51	19.65	+++
	SFRP1	-19.08	-2691	-498.7	10.56	-10.34	26.07	+++
	SFRP2	-23.25	-3270	-481	9.37	-11.56	3.31	++++
	SFRP3	9.94	-3561	-612.9	11.28	-8.99	254.86	+
	SFRP4	-10.41	-3016	-545.9	17.13	-11.87	1.98	++++
	SFRP5	-9.28	-3447	-606.5	9.78	-9.67	80.33	++
Wnt7a	Fzd1	-2.64	-3136	-542.9	10.84	-9.68	80.19	++
	Fzd2	3.5	-3493	-673.8	10.12	-8.25	893.79	-
	Fzd3	-8.89	-3080	-541.5	6.81	-8.77	372.24	+
	Fzd4	-20.11	-3111	-535.5	11.26	-11.01	8.35	++++
	Fzd5	-15.68	-3442	-548.7	10.40	-10.94	9.54	++++
	Fzd6	4.37	-3070	-506.4	6.78	-8.24	901.22	-
	Fzd7	-22.57	-3220	-567.7	9.83	-10.58	17.27	+++
	Fzd8	-16.27	-3406	-526.8	11.28	-11.42	4.18	++++
	Fzd9	-9.21	-2922	-492.3	12.16	-10.68	14.61	+++
	Fzd10	-3.59	-3292	-524.5	13.49	-11.06	7.73	++++
	SFRP1	-7.26	-2832	-496.8	10.56	-9.84	61.00	++

	SFRP2	-21	-3381	-531.1	11.15	-11.61	3.07	++++
	SFRP3	-4.47	-2918	-439.6	10.73	-10.51	19.50	+++
	SFRP4	-6.11	-2956	-514.9	15.77	-11.40	4.39	++++
	SFRP5	-15.36	-2835	-483.6	6.26	-9.22	174.30	+
Wnt7b	Fzd1	-3.94	-3354	-547.7	10.75	-10.10	39.30	+++
	Fzd2	-8.22	-3035	-534.7	9.71	-9.60	90.60	++
	Fzd3	-10.46	-2842	-507.1	5.46	-8.39	703.79	-
	Fzd4	-13.8	-3300	-603.7	10.03	-9.80	64.59	++
	Fzd5	-10.16	-3253	-585.5	9.40	-9.48	111.05	+
	Fzd6	12.78	-3149	-564.9	9.59	-8.03	1285.57	-
	Fzd7	-18.29	-3077	-547.4	10.29	-10.39	23.86	+++
	Fzd8	-13.94	-3074	-506	12.12	-11.13	6.91	++++
	Fzd9	2.98	-3194	-598.6	12.22	-9.21	177.08	+
	Fzd10	-4.48	-3568	-602.7	13.25	-10.69	14.41	+++
	SFRP1	-6.21	-2745	-438.8	8.30	-9.56	97.10	++
	SFRP2	-2.46	-2998	-401.5	9.54	-10.59	16.99	+++
	SFRP3	-9.73	-2881	-494.9	10.27	-10.03	44.03	++
	SFRP4	-4.33	-2914	-489.3	16.57	-11.73	2.49	++++
	SFRP5	-5.21	-2848	-484	12.08	-10.34	25.94	+++
Wnt8a	Fzd1	-10.91	-3078	-507.5	10.69	-10.47	20.86	+++
	Fzd2	-10.49	-3367	-569.5	11.62	-10.59	17.23	+++
	Fzd3	5.03	-2913	-450.2	6.81	-8.54	542.84	-
	Fzd4	-7.67	-3158	-512.2	10.10	-10.17	34.56	+++
	Fzd5	-17.51	-3342	-522.5	12.94	-11.94	1.74	++++
	Fzd6	-0.72	-2904	-507	6.89	-8.30	824.13	-
	Fzd7	-11.27	-3159	-517.1	11.42	-10.77	12.65	+++
	Fzd8	-28.02	-3586	-527.1	13.40	-13.21	0.21	++++
	Fzd9	-22.41	-2931	-431.5	12.48	-12.37	0.84	++++
	Fzd10	-13.34	-3076	-461	11.61	-11.44	4.07	++++
	SFRP1	-24.56	-2647	-492.2	10.29	-10.62	16.37	+++
	SFRP2	-21.24	-2994	-489.8	9.50	-10.84	11.23	+++
	SFRP3	-2.91	-3020	-492	11.43	-10.23	31.59	+++
	SFRP4	-13.24	-3193	-474.9	16.69	-13.06	0.26	++++
	SFRP5	-15.89	-2757	-503.8	7.37	-9.22	174.17	+
Wnt8b	Fzd1	-7.58	-3225	-528.3	10.95	-10.38	24.59	+++
	Fzd2	-4.29	-3430	-609.9	11.02	-9.65	83.80	++
	Fzd3	-3.05	-2935	-495.4	8.07	-9.00	248.78	+
	Fzd4	13.83	-3169	-530.3	10.56	-8.69	424.86	-
	Fzd5	-17.46	-2962	-497.2	11.95	-11.20	6.15	++++
	Fzd6	7.97	-2907	-452.8	9.66	-9.18	185.57	+
	Fzd7	-13.13	-3032	-596.3	10.07	-9.34	140.50	+
	Fzd8	-15.86	-3101	-521.5	12.47	-11.24	5.71	++++
	Fzd9	-15.52	-3457	-574.2	12.09	-11.19	6.25	++++
	Fzd10	-10	-3267	-547.4	12.82	-10.98	8.87	++++
	SFRP1	-7.7	-2670	-458	10.10	-9.85	59.23	++
	SFRP2	-18.02	-2781	-458.4	8.86	-10.37	24.64	+++
	SFRP3	-0.64	-2959	-440.4	11.37	-10.52	19.23	+++
	SFRP4	-4.71	-3294	-486.9	16.97	-12.63	0.54	++++
	SFRP5	-13.51	-2626	-466.9	10.42	-10.16	35.50	+++
Wnt9a	Fzd1	-3.09	-3155	-503.4	10.83	-10.18	33.99	+++
	Fzd2	-12.02	-3243	-598.4	9.97	-9.62	88.51	++

	Fzd3	-13.94	-3085	-541.8	6.34	-8.97	265.57	+
	Fzd4	13.28	-3553	-610.4	10.94	-8.67	435.30	-
	Fzd5	-2.71	-3263	-521	11.75	-10.45	21.72	+++
	Fzd6	10.62	-2962	-478.6	10.06	-8.94	277.98	+
	Fzd7	-15.49	-3546	-613	10.38	-10.39	23.98	+++
	Fzd8	-8.48	-3177	-487.3	12.30	-11.22	5.88	++++
	Fzd9	2.28	-3033	-479.1	11.77	-10.15	35.69	+++
	Fzd10	-3.81	-3260	-511.6	13.62	-11.20	6.11	++++
	SFRP1	-16.57	-2608	-408.5	9.20	-10.61	16.42	+++
	SFRP2	-10.72	-2436	-405.1	12.02	-10.80	12.08	+++
	SFRP3	-2.79	-3019	-456.7	10.67	-10.38	24.29	+++
	SFRP4	-15.6	-3350	-507.6	17.58	-13.42	0.14	++++
	SFRP5	-13.74	-2883	-402.1	12.16	-11.93	1.77	++++
Wnt9b	Fzd1	-14.64	-3079	-476.7	11.55	-11.34	4.84	++++
	Fzd2	-6.48	-3572	-574.4	10.85	-10.41	22.99	+++
	Fzd3	-2.98	-2969	-479.4	7.11	-8.95	273.20	+
	Fzd4	6.98	-3185	-535.9	11.98	-9.55	98.86	++
	Fzd5	-9.64	-3260	-514.7	11.05	-10.76	12.75	+++
	Fzd6	7.89	-3010	-479.1	9.73	-9.11	209.46	+
	Fzd7	-12.01	-3384	-551.5	10.45	-10.56	17.92	+++
	Fzd8	-3.27	-3351	-490.9	12.49	-11.22	5.86	++++
	Fzd9	-5.52	-3070	-501.6	12.06	-10.58	17.30	+++
	Fzd10	7.6	-3179	-488.6	12.84	-10.30	28.02	+++
	SFRP1	-7.42	-2773	-451.9	10.83	-10.32	26.78	+++
	SFRP2	-10.59	-2746	-425.1	8.94	-10.21	32.55	+++
	SFRP3	0.59	-3069	-436.2	10.13	-10.32	27.10	+++
	SFRP4	-8.43	-3264	-485.7	16.20	-12.60	0.57	++++
	SFRP5	-5.71	-3044	-443.9	11.02	-10.88	10.53	+++
Wnt10a	Fzd1	-17.53	-3085	-527.7	11.91	-11.08	7.49	++++
	Fzd2	-8.3	-3029	-570.3	11.67	-9.80	65.21	++
	Fzd3	-9.69	-2617	-460.4	9.21	-9.59	92.93	++
	Fzd4	2.16	-3453	-619	11.11	-9.18	184.02	+
	Fzd5	-10.49	-3182	-517.8	11.79	-10.87	10.71	+++
	Fzd6	4.99	-2820	-516.7	9.72	-8.51	573.00	-
	Fzd7	-14.7	-3030	-517.5	7.91	-9.67	80.93	++
	Fzd8	-11.98	-3220	-484.2	12.34	-11.59	3.18	++++
	Fzd9	-9.82	-3697	-646	12.71	-10.64	15.69	+++
	Fzd10	-20.91	-3142	-552.3	12.48	-11.31	5.07	++++
	SFRP1	-19.45	-2613	-443.5	8.38	-10.17	34.75	+++
	SFRP2	-19.4	-2894	-459.4	9.97	-11.01	8.35	++++
	SFRP3	-5.92	-3138	-541.8	9.80	-9.59	92.29	++
	SFRP4	-24.51	-3024	-550.2	16.64	-12.63	0.54	++++
	SFRP5	-11.94	-3029	-511.8	9.26	-9.96	49.44	++
Wnt10b	Fzd1	-18.67	-3097	-511.1	10.60	-10.96	9.11	++++
	Fzd2	-12.81	-2974	-476	9.97	-10.54	18.71	+++
	Fzd3	-10.09	-2761	-505.9	6.78	-8.63	468.54	-
	Fzd4	16.56	-3172	-570.9	12.01	-8.50	584.60	-
	Fzd5	-11.08	-3019	-544.6	11.42	-10.18	34.37	+++
	Fzd6	-6.25	-3088	-522.9	9.74	-9.72	74.96	++
	Fzd7	-23.69	-2802	-462.3	11.05	-11.42	4.19	++++
	Fzd8	-20.72	-2926	-478.8	12.60	-11.75	2.40	++++

	Fzd9	-12.91	-3336	-578.7	11.96	-10.69	14.51	+++
	Fzd10	-11.45	-3071	-570.8	13.15	-10.54	18.68	+++
	SFRP1	-20.11	-2568	-449.2	8.72	-10.17	34.94	+++
	SFRP2	-14.48	-2731	-499.5	8.32	-9.41	125.10	+
	SFRP3	-4.21	-3421	-603.5	10.13	-9.43	122.19	+
	SFRP4	-0.46	-3283	-578	15.86	-10.96	9.19	++++
	SFRP5	-16.14	-2957	-466.6	8.28	-10.31	27.32	+++
Wnt11	Fzd1	-10.95	-2987	-458.2	11.15	-11.00	8.55	++++
	Fzd2	0.91	-3476	-665.6	10.24	-8.52	563.89	-
	Fzd3	2.67	-2729	-435.4	5.95	-8.25	888.85	-
	Fzd4	5.21	-3519	-645.2	11.35	-8.89	304.40	+
	Fzd5	-5.3	-3341	-586.3	10.09	-9.53	103.02	+
	Fzd6	2.14	-3143	-530	6.11	-8.06	1226.50	-
	Fzd7	-3.72	-2953	-528.8	9.68	-9.20	178.41	+
	Fzd8	-16.77	-3175	-504.7	11.79	-11.42	4.19	++++
	Fzd9	-0.16	-3174	-560.8	12.17	-9.79	66.13	++
	Fzd10	-5.19	-3597	-603.7	13.34	-10.81	11.80	+++
	SFRP1	-8.59	-2501	-400.6	10.54	-10.38	24.59	+++
	SFRP2	-20.53	-2861	-525.4	10.90	-10.57	17.80	+++
	SFRP3	3.45	-2816	-489.7	10.57	-9.17	187.56	+
	SFRP4	-11.64	-3063	-451.2	15.29	-12.54	0.63	++++
	SFRP5	-10.96	-3051	-528.5	8.93	-9.65	84.03	++
Wnt16	Fzd1	-14.11	-3099	-518.3	9.93	-10.37	24.72	+++
	Fzd2	-12.17	-3447	-608.3	10.32	-10.01	45.21	++
	Fzd3	-21.33	-3251	-593.3	6.05	-9.11	207.79	+
	Fzd4	7.73	-3386	-633.1	12.80	-9.04	234.00	+
	Fzd5	-14.61	-3184	-584.7	11.18	-10.21	32.76	+++
	Fzd6	9.65	-3306	-586	10.07	-8.45	630.14	-
	Fzd7	-23.3	-2965	-530	10.29	-10.71	13.91	+++
	Fzd8	-17.36	-3113	-487	11.34	-11.41	4.32	++++
	Fzd9	-9.06	-3515	-604.6	10.67	-10.08	40.19	++
	Fzd10	-21.17	-3255	-568.2	13.01	-11.53	3.50	++++
	SFRP1	-10.4	-2599	-477.4	6.49	-8.57	517.66	-
	SFRP2	-4.92	-3171	-540.7	12.18	-10.33	26.44	+++
	SFRP3	-4.86	-2951	-517.7	12.05	-10.13	37.39	+++
	SFRP4	-11.02	-3059	-568.1	13.25	-10.55	18.41	+++
	SFRP5	-8.94	-2793	-485.8	10.21	-9.89	55.40	++

[a]ΔG is in kcal/mol and calculated according to Model 1: ΔG = 0.06715 × CP_TSC + 0.001913 × CP_ELOCAL_CB – 0.01128 × CP_ELOCAL_MIN – 0.3072 × MMGBSA dG Bind Solv GB – 6.2941. K_d is in nM and calculated according to the following equation: $K_d = e^{\frac{\Delta G}{RT}} \times 10^9$, where ΔG is the binding energy predicted by the new model, R is the gas constant (1.987×10^{-3} kcal/(K mol)) and T is standard ambient temperature (298K). Approximate strength based on ranges defined by Dijksterhuis et al. (1): ++++, <10nM; +++, 10-40nM; ++, 40-100nM; +, 100-400nM; -, >400nM.

Table S4. Descriptor values, predicted binding energy and predicted dissociation constants for all Wnt-cryptic Fzd CRD interactions.

PROTEINS		DESCRIPTORS				BINDING AFFINITY		
Wnt	CRD	CP_TSC	CP_ELOCAL _CB	CP_ELOCAL _MIN	MMGBSA dG Bind Solv GB	ΔG	K_d	Approx Strength
Wnt1	COL18A1	2.7	-3028	-508.3	8.40	-8.75	381.54	+
	CORIN Fz1	-17.41	-3243	-574.4	12.22	-10.94	9.46	++++
	CORIN Fz2	-10.4	-3023	-427.1	8.55	-10.58	17.27	+++
	CPZ	2.88	-2522	-444.8	10.19	-9.04	235.06	+
	MFRP	-25.68	-2744	-477.1	9.48	-10.80	12.03	+++
	MuSK	-3.65	-3637	-555.2	12.86	-11.18	6.27	++++
	ROR1	10.68	-3390	-576.4	13.23	-9.62	87.71	++
	ROR2	-34.72	-3084	-502	11.38	-12.36	0.87	++++
	SMO	-0.02	-2899	-546	8.09	-8.17	1021.79	-
Wnt2	COL18A1	18.47	-3019	-400.9	8.50	-8.92	288.18	+
	CORIN Fz1	-11.46	-3487	-534.8	4.91	-9.21	176.40	+
	CORIN Fz2	4.68	-3389	-487.5	3.99	-8.19	987.84	-
	CPZ	-4.11	-2421	-355.8	8.25	-9.72	74.13	++
	MFRP	-15.7	-3235	-373.5	11.91	-12.98	0.30	++++
	MuSK	-17.04	-3678	-449.3	19.18	-15.30	0.01	++++
	ROR1	-10.9	-3645	-631.8	10.78	-10.18	34.03	+++
	ROR2	-3.74	-3591	-649	10.90	-9.44	119.09	+
	SMO	-0.56	-3075	-480.7	8.45	-9.39	130.31	+
Wnt2b	COL18A1	6.57	-2576	-378.1	5.92	-8.33	772.17	-
	CORIN Fz1	-1.36	-3087	-495.7	6.10	-8.57	515.65	-
	CORIN Fz2	-14.83	-3853	-699.8	8.05	-9.24	167.73	+
	CPZ	-0.69	-2516	-374.9	7.27	-9.16	191.96	+
	MFRP	-11.19	-2905	-385.7	6.89	-10.37	24.86	+++
	MuSK	-15.89	-3282	-419	17.23	-14.20	0.04	++++
	ROR1	-3.32	-3675	-553.7	14.69	-11.81	2.17	++++
	ROR2	-13.35	-3625	-574.8	6.33	-9.59	93.27	++
	SMO	1.49	-2968	-499.7	9.06	-9.02	243.14	+
Wnt3	COL18A1	8.02	-2907	-428.7	6.81	-8.57	515.42	-
	CORIN Fz1	-32.54	-3441	-569.2	8.79	-11.34	4.81	++++
	CORIN Fz2	-10.69	-3447	-538.4	7.92	-9.97	49.15	++
	CPZ	-0.19	-2470	-415.4	8.56	-8.97	262.15	+
	MFRP	-18.48	-2803	-348.5	11.24	-12.42	0.78	++++
	MuSK	0.81	-3197	-458.8	16.75	-12.32	0.91	++++
	ROR1	-9.71	-3155	-544.9	7.00	-8.99	256.87	+
	ROR2	-19.02	-3778	-677.2	8.09	-9.64	84.65	++
	SMO	-1.93	-2990	-507.6	9.01	-9.19	183.28	+
Wnt3a	COL18A1	14.88	-3276	-525.5	7.23	-7.85	1734.50	-
	CORIN Fz1	-15.09	-3105	-480.2	5.63	-9.56	97.48	++
	CORIN Fz2	-9.56	-3285	-522	7.77	-9.72	74.73	++
	CPZ	0.26	-2413	-453.4	9.63	-8.74	391.68	+
	MFRP	-20.34	-2425	-354.1	11.35	-11.79	2.26	++++

	MuSK	-15.28	-3416	-468.6	18.40	-14.22	0.04	++++
	ROR1	-7.67	-3030	-488.7	11.43	-10.60	16.72	+++
	ROR2	-29.2	-3437	-580	9.37	-11.16	6.49	++++
	SMO	-7.91	-3050	-532.5	11.02	-10.04	43.40	++
Wnt4	COL18A1	12.64	-2857	-434.9	7.43	-8.29	835.27	-
	CORIN Fz1	-14.71	-3444	-584.8	7.04	-9.43	120.43	+
	CORIN Fz2	-10.36	-3724	-560.1	7.23	-10.02	45.04	++
	CPZ	-11.19	-2332	-341.9	4.47	-9.02	241.98	+
	MFRP	-7.76	-2684	-428.4	12.22	-10.87	10.63	+++
	MuSK	-9.9	-3630	-539	14.26	-12.20	1.12	++++
	ROR1	-1.82	-3625	-536.3	12.17	-11.04	8.02	++++
	ROR2	-31.83	-3860	-648.2	12.45	-12.33	0.91	++++
	SMO	2.16	-2882	-495.7	9.32	-8.93	281.39	+
Wnt5a	COL18A1	6.67	-2789	-428.3	4.03	-7.59	2720.79	-
	CORIN Fz1	-6.93	-3293	-497	9.86	-10.48	20.56	+++
	CORIN Fz2	3.28	-3187	-505.8	7.58	-8.79	355.32	+
	CPZ	-4.15	-2184	-359.7	10.08	-9.79	66.16	++
	MFRP	-26.14	-2913	-461.9	11.40	-11.91	1.83	++++
	MuSK	-9.69	-3791	-576.8	11.81	-11.32	5.01	++++
	ROR1	-12.31	-3346	-553.1	11.46	-10.80	11.96	+++
	ROR2	-31.95	-3499	-567.2	16.54	-13.81	0.07	++++
	SMO	6.87	-2657	-469.6	8.76	-8.31	806.76	-
Wnt5b	COL18A1	9.26	-2962	-432.8	4.24	-7.76	2042.55	-
	CORIN Fz1	-11.43	-3509	-554.5	9.62	-10.47	20.80	+++
	CORIN Fz2	-8.74	-3271	-506	8.01	-9.89	55.62	++
	CPZ	1.92	-2580	-474	10.05	-8.84	328.83	+
	MFRP	-13.53	-2802	-415.7	11.81	-11.50	3.67	++++
	MuSK	-8.1	-3532	-485	20.65	-14.47	0.02	++++
	ROR1	-6.25	-3346	-524.1	11.23	-10.65	15.42	+++
	ROR2	-28.12	-3938	-614.8	8.65	-11.44	4.09	++++
	SMO	4.35	-2994	-573.3	10.09	-8.36	736.10	-
Wnt6	COL18A1	-2.49	-3143	-505.3	5.54	-8.47	609.45	-
	CORIN Fz1	-8.64	-3642	-622.3	5.98	-8.66	447.78	-
	CORIN Fz2	-6.08	-3647	-618	7.74	-9.09	217.12	+
	CPZ	3.31	-2504	-442.3	10.23	-9.01	245.16	+
	MFRP	-20.68	-2768	-395	11.22	-11.97	1.67	++++
	MuSK	-16.2	-4084	-436.4	16.68	-15.39	0.01	++++
	ROR1	-6.08	-3518	-524.1	14.77	-12.06	1.43	++++
	ROR2	-30.45	-3438	-596	9.41	-11.08	7.44	++++
	SMO	12.73	-3275	-631.8	9.51	-7.50	3174.24	-
Wnt7a	COL18A1	6.35	-3130	-425.9	5.83	-8.84	327.90	+
	CORIN Fz1	-17.38	-3229	-485.3	4.48	-9.54	101.07	+
	CORIN Fz2	-15.05	-3674	-497.6	6.81	-10.81	11.81	+++
	CPZ	5.45	-2403	-396.7	9.32	-8.91	290.70	+
	MFRP	-12.64	-2997	-350.5	11.08	-12.33	0.91	++++
	MuSK	-13.75	-3838	-555	16.06	-13.23	0.20	++++
	ROR1	-0.63	-3628	-554	11.72	-10.63	16.06	+++

	ROR2	-27.68	-3841	-575.8	10.02	-12.08	1.38	++++
	SMO	2.99	-2874	-463.6	6.89	-8.48	605.52	-
Wnt7b	COL18A1	12.14	-2896	-472.6	8.29	-8.23	915.27	-
	CORIN Fz1	-10.34	-3076	-451.3	6.79	-9.87	58.11	++
	CORIN Fz2	-14.01	-3231	-490	8.35	-10.45	21.57	+++
	CPZ	3.46	-2563	-415	8.43	-8.87	310.27	+
	MFRP	-17.28	-2847	-447.8	10.47	-11.06	7.68	++++
	MuSK	-8.48	-3504	-475.6	19.85	-14.30	0.03	++++
	ROR1	-23.4	-3341	-529.2	9.15	-11.10	7.27	++++
	ROR2	-28.71	-3526	-570.1	11.41	-12.04	1.47	++++
	SMO	7.74	-3025	-513.9	7.19	-7.97	1422.44	-
Wnt8a	COL18A1	7.89	-3079	-492.1	5.75	-7.87	1690.50	-
	CORIN Fz1	-11.81	-3696	-545.3	11.29	-11.47	3.84	++++
	CORIN Fz2	-6.04	-3418	-513.5	7.66	-9.80	65.30	++
	CPZ	-11.85	-2243	-351.7	10.10	-10.52	19.36	+++
	MFRP	-12.14	-2953	-438.3	7.49	-10.11	38.16	+++
	MuSK	-17.43	-3606	-452.3	13.50	-13.41	0.15	++++
	ROR1	-12.83	-3482	-611.8	7.09	-9.09	214.52	+
	ROR2	-19.95	-3759	-564.4	11.80	-12.08	1.38	++++
	SMO	-4.62	-2811	-477.6	9.56	-9.53	102.64	+
Wnt8b	COL18A1	2.21	-2804	-417.4	3.96	-8.02	1321.23	-
	CORIN Fz1	-4.82	-3617	-615.6	10.22	-9.73	73.03	++
	CORIN Fz2	-0.7	-3463	-553.8	7.59	-9.05	230.42	+
	CPZ	-4.71	-2338	-416.5	7.31	-8.63	468.76	-
	MFRP	-18.44	-2645	-357.5	9.89	-11.60	3.13	++++
	MuSK	-10.54	-3465	-464.6	16.85	-13.56	0.11	++++
	ROR1	-23.74	-3582	-619.5	8.72	-10.43	22.45	+++
	ROR2	-24.49	-3453	-523.2	11.54	-12.19	1.15	++++
	SMO	1.86	-2879	-451.2	9.62	-9.54	100.67	+
Wnt9a	COL18A1	8.75	-3001	-468.1	8.33	-8.72	399.44	+
	CORIN Fz1	-14.76	-3802	-544.5	6.15	-10.30	27.76	+++
	CORIN Fz2	-5.54	-3937	-577.8	1.63	-8.18	999.89	-
	CPZ	-9.12	-2520	-394.8	7.62	-9.61	89.00	++
	MFRP	-16.74	-2959	-364.1	11.85	-12.61	0.56	++++
	MuSK	1.56	-3565	-418.4	16.76	-13.44	0.14	++++
	ROR1	5.66	-3966	-639.1	11.79	-9.91	53.56	++
	ROR2	-14.21	-3339	-431.1	12.58	-12.64	0.54	++++
	SMO	2.38	-3150	-590	8.00	-7.96	1444.97	-
Wnt9b	COL18A1	10.42	-3111	-427	5.81	-8.51	571.76	-
	CORIN Fz1	1.21	-3687	-573.5	5.93	-8.62	477.83	-
	CORIN Fz2	-0.51	-3831	-557.6	6.90	-9.49	110.54	+
	CPZ	-5.71	-2670	-403.2	7.26	-9.47	113.88	+
	MFRP	-8.89	-3055	-467.7	10.19	-10.59	17.17	+++
	MuSK	-8.87	-3910	-492.1	12.46	-12.65	0.53	++++
	ROR1	-13.76	-3587	-616.6	6.94	-9.26	162.85	+
	ROR2	-29.39	-3832	-512.4	11.44	-13.33	0.17	++++
	SMO	-0.69	-3401	-572.9	8.44	-8.98	260.76	+

Wnt10a	COL18A1	19.58	-2965	-418.4	4.26	-7.24	4901.39	-
	CORIN Fz1	-17.4	-3150	-526.5	13.06	-11.56	3.32	++++
	CORIN Fz2	5.84	-3638	-641.7	2.23	-6.31	23697.99	-
	CPZ	4.94	-2445	-470.5	9.42	-8.23	926.82	-
	MFRP	-11.72	-2737	-417.7	12.13	-11.33	4.90	++++
	MuSK	-16.29	-3811	-567.1	14.26	-12.66	0.52	++++
	ROR1	-17.29	-3233	-533.2	11.66	-11.21	6.04	++++
	ROR2	-16.02	-3431	-554.7	7.94	-10.11	38.24	+++
	SMO	7.33	-2755	-484.2	6.42	-7.58	2755.43	-
Wnt10b	COL18A1	10.57	-2749	-413.3	5.52	-7.88	1669.22	-
	CORIN Fz1	-27.93	-3146	-459	4.87	-10.51	19.73	+++
	CORIN Fz2	-10.93	-3228	-492.9	7.83	-10.05	42.65	++
	CPZ	-5.91	-2372	-416.1	8.59	-9.17	187.18	+
	MFRP	-7.76	-2392	-351	11.68	-11.02	8.29	++++
	MuSK	-14.37	-3280	-479.6	18.82	-13.90	0.06	++++
	ROR1	-8.38	-3079	-506.1	8.66	-9.70	77.06	++
	ROR2	-27.16	-3153	-436.9	11.45	-12.74	0.45	++++
	SMO	0.17	-2868	-542.9	9.07	-8.43	656.02	-
Wnt11	COL18A1	6.96	-3083	-549.1	3.96	-6.75	11251.47	-
	CORIN Fz1	-3.11	-3361	-526.9	11.89	-10.64	15.69	+++
	CORIN Fz2	2.59	-3301	-521.4	7.72	-8.92	285.11	+
	CPZ	-13.87	-2371	-424	8.75	-9.67	81.44	++
	MFRP	-16.41	-2840	-478.2	9.14	-10.24	30.81	+++
	MuSK	-5.72	-3786	-543.3	13.61	-11.97	1.66	++++
	ROR1	-3.32	-3208	-518.7	8.51	-9.42	124.38	+
	ROR2	-14.34	-3171	-480.3	10.18	-11.03	8.11	++++
	SMO	4.88	-3432	-592.3	9.96	-8.91	292.26	+
Wnt16	COL18A1	16.83	-3208	-476.1	7.28	-8.16	1028.07	-
	CORIN Fz1	-18.05	-3544	-530.7	4.53	-9.69	78.35	++
	CORIN Fz2	-7.97	-3642	-613.5	4.02	-8.11	1127.78	-
	CPZ	-12.03	-2421	-397.6	4.64	-8.67	436.39	-
	MFRP	-21.35	-2803	-382.1	10.23	-11.92	1.81	++++
	MuSK	-14.52	-3653	-520.7	18.00	-13.91	0.06	++++
	ROR1	-13.06	-3454	-570.5	9.55	-10.28	28.99	+++
	ROR2	-36.26	-3548	-565.4	9.63	-12.10	1.34	++++
	SMO	-11.62	-3115	-542.6	8.26	-9.45	117.33	+

[a]ΔG is in kcal/mol and calculated according to Model 1: ΔG = 0.06715 × CP_TSC + 0.001913 × CP_ELOCAL_CB – 0.01128 × CP_ELOCAL_MIN – 0.3072 × MMGBSA dG Bind Solv GB – 6.2941. K_d is in nM and calculated according to the following equation: $K_d = e^{\frac{\Delta G}{RT}} \times 10^9$, where ΔG is the binding energy predicted by the new model, R is the gas constant (1.987 × 10-3 kcal/(K mol)) and T is standard ambient temperature (298K). Approximate strength based on ranges defined by Dijksterhuis et al. (1): ++++, <10nM; +++, 10-40nM; ++, 40-100nM; +, 100-400nM; -, >400nM.

Table S5. Aligned sequences of Class F GPCR intracellular loops (ICLs).[a]

Protein	ICL1	ICL2	ICL3
SMO	DWRNSNRY	TYAWHTSFKALGTTYQPLSGKTS	MTLFSIKSNHPGLLSEKAASKINETMLR
Fzd1	DMRR-FSY	SLTWFLAAG-MKWGHEAIEANSQ	VSLFRIRTIMKH--DGTKTEKLEKLMVR
Fzd2	DMQR-FRY	SLTWFLAAG-MKWGHEAIEANSQ	VSLFRIRTIMKH--DGTKTEKLERLMVR
Fzd3	DVTR-FRY	TITWFLAAV-PKWGSEAIEKKAL	ISLNRVRIEIPL--EKENQDKLVKFMIR
Fzd4	DSSR-FSY	TLTWFLAAG-LKWGHEAIEMHSS	VALFKIRSNLQK--
Fzd5	DMER-FRY	SLTWFLAAG-	VSLFRIRSVIKQ--GGTKTDKLEKLMIR
Fzd6	DVRR-FRY	TITWFLAAG-RKWSCEAIEQKAV	ISLNHVRQVIQH--
Fzd7	DMRR-FSY	SLTWFLAAG-MKWGHEAIEANSQ	VSLFRIRTIMKH--DGTKTEKLEKLMVR
Fzd8	DMER-FKY	SLTWFLAAG-MKWGNEAIAGYSQ	VSLFRIRSVIKQQDGPTKTHKLEKLMIR
Fzd9	EPHR-FQY	TLTWFLAAG-KKWGHEAIEAHGS	VALFHIRKIMKT--
Fzd10	DPAR-FRY	TLTWFLAAG-KKWGHEAIEANSS	VALFHIRRVMKT--

[a]Human sequences identified and aligned using UniProt. Regions of SMO indicated as transmembrane in UniProt used to define limits of ICLs.
